# Mammals Achieve Common Neural Coverage of Visual Scenes Using Distinct Sampling Behaviors

**DOI:** 10.1523/ENEURO.0287-23.2023

**Published:** 2024-02-06

**Authors:** Jason M. Samonds, Martin Szinte, Carrie Barr, Anna Montagnini, Guillaume S. Masson, Nicholas J. Priebe

**Affiliations:** ^1^Center for Learning and Memory and the Institute for Neuroscience, The University of Texas at Austin, Austin 78712, Texas; ^2^Institut de Neurosciences de la Timone (UMR 7289), Centre National de la Recherche Scientifique and Aix-Marseille Université, 13385 Marseille, France

**Keywords:** fixation, natural scenes, saccades, visual cortex

## Abstract

Most vertebrates use head and eye movements to quickly change gaze orientation and sample different portions of the environment with periods of stable fixation. Visual information must be integrated across fixations to construct a complete perspective of the visual environment. In concert with this sampling strategy, neurons adapt to unchanging input to conserve energy and ensure that only novel information from each fixation is processed. We demonstrate how adaptation recovery times and saccade properties interact and thus shape spatiotemporal tradeoffs observed in the motor and visual systems of mice, cats, marmosets, macaques, and humans. These tradeoffs predict that in order to achieve similar visual coverage over time, animals with smaller receptive field sizes require faster saccade rates. Indeed, we find comparable sampling of the visual environment by neuronal populations across mammals when integrating measurements of saccadic behavior with receptive field sizes and V1 neuronal density. We propose that these mammals share a common statistically driven strategy of maintaining coverage of their visual environment over time calibrated to their respective visual system characteristics.

## Significance Statement

Mammals rapidly move their eyes to sample their visual environment over successive fixations, but they use different spatial and temporal strategies for this sampling. We demonstrate that these different strategies achieve similar neuronal receptive field coverage over time. Because mammals have distinct sensory receptive field sizes and neuronal densities for sampling and processing information, they require different eye movement strategies to encode natural scenes.

## Introduction

**Figure 1.**
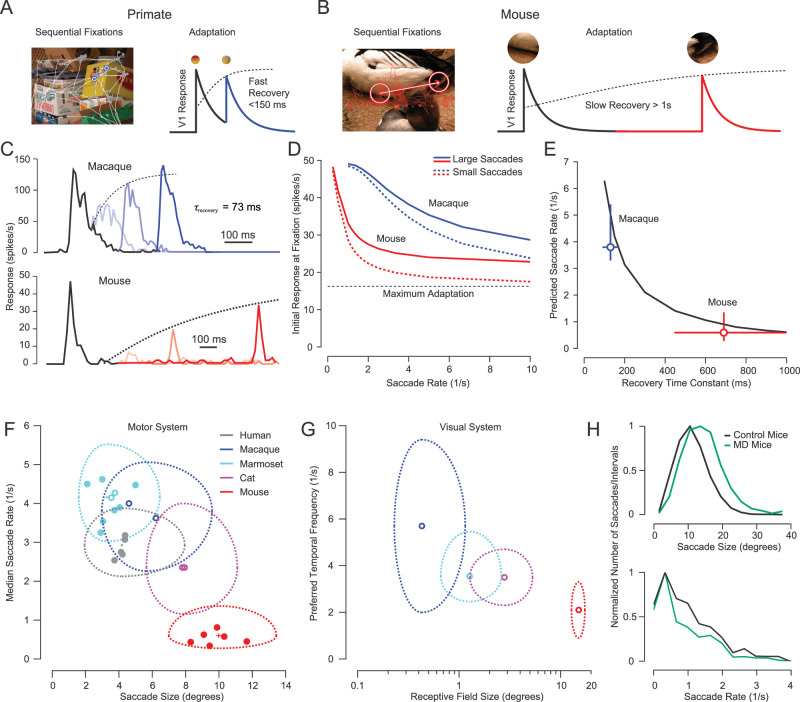
Saccade rates increase with decreasing saccade size matching faster recovery from adaptation for smaller receptive fields. ***A***, ***B***, Sequential fixations change receptive field inputs and we hypothesize that differences in adaptation recovery time constants would allow primates (***A***) to make more frequent saccades compared with mice (***B***). ***C***, Top, Example macaque MT responses to two repeated presentations of the same stimulus (Priebe et al., 2002). Summary statistics suggest that the dynamics of adaptation for macaque V1 neurons to repeated stimulation are similar to this example MT neuron (Patterson et al., 2013). Black represents neural activity to the first presentation and different shades of blue depict the neural activity after the second presentation in progressively more delayed conditions. Bottom, Example mouse V1 responses to a similar sequential experiment (Jin and Glickfeld, 2020). See model example responses in Extended Data Fig. 1-1. ***D***, Magnitude of initial model responses for different saccade rates for macaques and mice. To mimic large saccades, the stimulus changed randomly after a saccade (decorrelated), while to mimic small saccades, the stimulus stayed the same after a saccade. ***E***, Saccade rates predicted for a range of recovery time constants that maintain 75% of initial model responses (black). Blue vertical error bars are 25th and 75th percentile quartiles of observed saccade sizes from Mitchell et al. (2014), and horizontal error bars are the 95% confidence interval of recovery time constants measured by Patterson et al. (2013). Red vertical error bars are 25th and 75th percentile quartiles of observed saccade sizes from Samonds et al. (2018), and horizontal error bars are the 95% confidence interval of recovery time constants measured by Jin and Glickfeld (2020). ***F***, Saccade rate versus size distributions for five species. Each solid point represents the medians of a single subject. Open circles of marmosets (Mitchell et al., 2014), macaques (Mitchell et al., 2014), and cats represent previously published data from other laboratories (see Materials and Methods). The large dashed outline represents the central half of the distribution (between the 25th and 75th percentiles), and the small cross in the center represents the standard error of the median for the distribution of all subjects. Due to the large number of samples, the standard error bars are smaller than even the size of the data points. See full distributions in Extended Data Fig. 1-2. Marmosets naturally use their heads for nearly all changes in gaze leading to larger saccade sizes than shown in ***F*** (Movies 1 and 2 and Extended Data Fig. 1-4 and 1-5). ***G***, Preferred temporal frequency versus receptive field size distributions from previously published data (see Materials and Methods). The open circle represents the median, and the large dashed outline represents the central half of the distribution (between the 25th and 75th percentiles). Contrast-dependent reductions in preferred temporal frequency correlate with reduced saccade rates (Extended Data Figs. 1-3). ***H***, Saccade size increases (Samonds et al., 2018) and saccade rate decreases in mice with MD during the critical period (*N* = 4 mice; *n* = 1,448 saccades) compared with control mice (*N* = 3 mice; *n* = 1586 saccades).

A large body of work clearly shows that we move our eyes to align the central portion of our visual field on goal-relevant objects (Yarbus, 1967; Koch and Ullman, 1985; Hayhoe and Ballard, 2005; Najemnik and Geisler, 2005; Gegenfurtner, 2016), but even when we are not engaged in a task or even when we are fixating on an object, we incessantly continue to make saccadic eye movements several times a second. Outside of highly controlled experimental setups (Najemnik and Geisler, 2005), the target of many of our gaze changes are not predictable (Schutz et al., 2011). Similar to humans, many other vertebrates also view the world over a sequence of discrete stable fixations generated from coordinated head and eye movements (Walls, 1962; Land, 1999, 2019). The distributions of size and frequency of the gaze changes between fixations vary substantially for different mammals, including different nonhuman primates (Samonds et al., 2018). Even during passive viewing and irrespective of the presence of a fovea, gaze changes are informative by overcoming inhomogeneity in the sensory representation of different animals and providing receptive fields a unique and updated perspective at each fixation (Fig. 1*a*,*b*). The retina in several animals contains a differential density of photoreceptors (Rapaport and Stone, 1984; Szel et al., 1992; Goodchild et al., 1996; Jeon et al., 1998) and occlusion by retinal vasculature (Schiefer et al., 1999). This inhomogeneity persists along visual pathways and is related to cortical magnification factor (Wassle et al., 1989; Chaplin et al., 2013), acuity (Albus, 1975; Wilson and Sherman, 1976; Tusa et al., 1978; Van Essen et al., 1984; van Beest et al., 2021; Tan et al., 2022), color sensitivity (Rhim et al., 2017), and irregular ocular dominance domains (Adams and Horton, 2002). Additional inhomogeneities emerge within cortex, such as the irregular distribution of orientation representation in mice (Tan et al., 2022) and disparity selectivity in mice and nonhuman primates (Sprague et al., 2015; La Chioma et al., 2019; Samonds et al., 2019). By making gaze changes to cover different regions of the scene, an internal representation may be constructed by integrating novel receptive field information over successive fixations (Gottlieb, 2007; Melcher and Colby, 2008; Cavanagh et al., 2010; Ganmor et al., 2015; Wolf and Schutz, 2015; Stewart et al., 2020).

10.1523/ENEURO.0287-23.2023.f1-1 Extended Data Figure 1-1 **Example V1 responses modeled with a divisive gain time constant** **A**, Example model macaque V1 responses to two repeated presentations of the same stimulus. Black represents neural activity to the first presentation and different shades of blue depict the neural activity after the second presentation in progressively more delayed conditions. **B**, Same format as a for mouse model V1 responses. Download Figure 1-1, TIF file.

**Figure 2.**
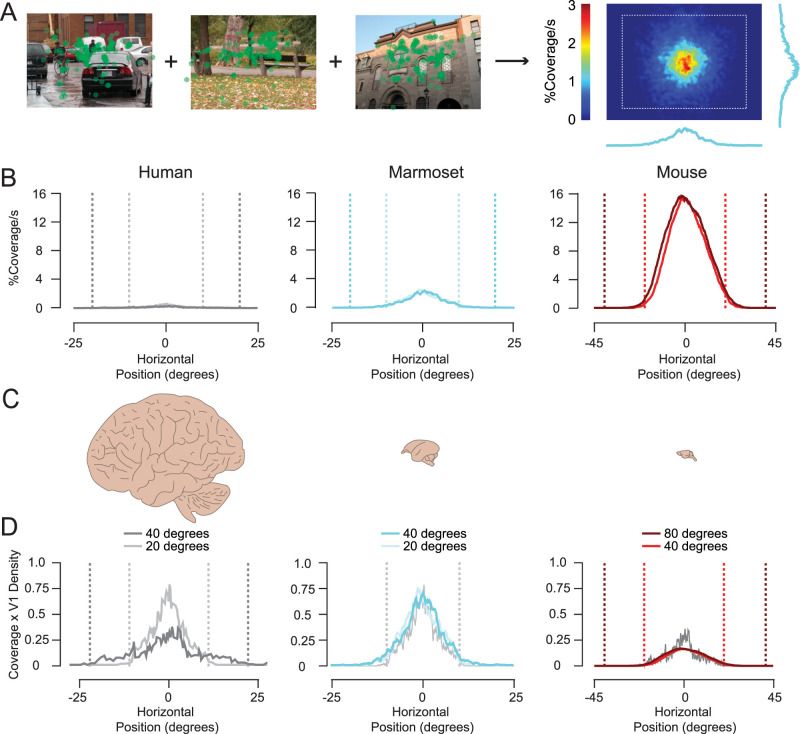
Fixations over time provide similar visual processing coverage across different mammals. ***A***, Computing average receptive field coverage over time from saccades. ***B***, Horizontal cross sections of receptive field coverage over time for each mammal. Lighter colors are data obtained when subjects were viewing small images (Extended Data Fig. 2-1). Vertical dashed lines represent image boundaries. ***C***, Relative brain sizes between each mammal. ***D***, Horizontal coverage data from ***B*** multiplied by the average two-dimensional V1 cell density for each mammal. Human data are replotted with marmoset and mouse data to facilitate comparison. Note that this is coverage based on decorrelating V1 receptive field responses, which have spatial frequency preferences (Samonds et al., 2018) that fall outside of the spectrum of whitening due to saccade and fixation dynamics (Mostofi et al., 2020; Extended Data Fig. 2-2).

10.1523/ENEURO.0287-23.2023.f1-2 Extended Data Figure 1-2 **Saccadic eye movement statistics while viewing natural images** **A**, Eye traces and fixation points (circles) for humans, marmosets and mice viewing an example large (top row) or small (bottom row) image for 30 s. For mice, we plotted fixation points with respect to an assumed projection of binocular photoreceptors by aligning the center of the image with the median eye position, since standard fixation calibration procedures are not possible. **B**, Histograms of saccade sizes for N = 5 humans each viewing 80 large (n = 49,907 saccades) and small (n = 42,523 saccades) images, N = 7 marmosets each viewing 24 large (n = 25,531 saccades) and small images (n = 24,781 saccades), and N = 6 mice each viewing 8-18 large (n = 1,355 saccades) and small (n = 1,145 saccades) images (Samonds et al. 2018). **C**, Histograms of saccade rates (1/intersaccadic interval) for the same subjects. Download Figure 1-2, TIF file.

10.1523/ENEURO.0287-23.2023.f1-3 Extended Data Figure 1-3 **Saccade rates decrease with decreasing contrast** **A**, Each solid point represents the medians of a single subject. The large outline represents 25th and 75th percentiles and the small outline represents the standard error of the median for the distribution of all subjects. Due to the large number of samples, the standard error of the median outlines are smaller than even the data points. Saccade rate versus size distributions for n = 5 humans for images with progressively reduced contrast (small and large images combined). (100%: n = 92,430 saccades; 10%: n = 42,767 saccades; 5%: n = 41,639 saccades). **B**, Same data for n = 5 marmosets. (100%: n = 40,474 saccades; 10%: n = 31,927 saccades; 5%: n = 37,294 saccades). **C**, Same data for n = 4 mice. (100%: n = 472 saccades; 50%: n = 338 saccades; 25%: n = 274 saccades). For all three animals, there was a clear decrease in saccade rate for the images with reduced contrast (from dark to light colors; bootstrapped, 5 and 10 versus 100%, p < 0.001 for all comparisons for humans and marmosets; 25 and 50 versus 100%, p = 0.008 and 0.01, respectively for mice). Download Figure 1-3, TIF file.

10.1523/ENEURO.0287-23.2023.f1-4 Extended Data Figure 1-4 **Gaze position over time for posted and unposted marmosets** **A**, Horizontal and vertical pupil position for both eyes for a head-fixed marmoset. Green circles are saccade onset and red circles are saccade offset. **B**, Same gaze data for the same marmoset now able to move their head freely unposted (head-free). Forehead position and left eye position relative to the head are included with the gaze traces. Note that the eyes never rotate out more than ±15 degrees and head movements drive the gaze changes. Download Figure 1-4, TIF file.

10.1523/ENEURO.0287-23.2023.f1-5 Extended Data Figure 1-5 **Contribution of head and eye movements to marmoset gaze changes** **A**, An example of a large change in gaze that is mostly driven by a saccadic head movement. Vestibular ocular reflex (VOR) movements at the beginning and end of the head movement lead to an overall faster gaze changes separated by two stable periods of fixation. **B**, An example of a small change in gaze. Although most of the movement is the result of the eyes, there is still a significant contributing saccadic head movement and there are similar VOR dynamics as those observed with a large change in gaze. **C**, Distributions of saccadic gaze change sizes for head fixed and freely moving (head-free) marmosets. **D**, Distributions of saccadic gaze change rates for head fixed and head-free marmosets. Download Figure 1-5, TIF file.

Our hypothesis is that neural adaptation and gaze changes work together to provide novel receptive field inputs and conserve energy. Neurons quickly adapt their responsiveness to unchanging visual input (Muller et al., 1999), which should lead to the conservation of metabolic energy by reducing overall neural activity (Sheth et al., 2004; Tring et al., 2023). Eye and head movements, which change where a receptive field samples, could be continuous and uniform across the visual scene, but that would be inefficient and prevent sufficient processing that requires a stable view of images (Land, 2019). To conserve energy and allow the nervous system to process visual inputs, gaze changes are discrete and quick only moving on average the minimum distance necessary to provide novel inputs to the receptive fields given natural scene statistics (Samonds et al., 2018). This argument successfully accounts for the differences in the distribution of saccade amplitudes across mammals (Samonds et al., 2018), but it is not clear if it also explains differences in saccade rates. Since sampling the visual environment depends on both space and time, we expanded our conceptual model to include adaptation dynamics, examined the differences in visual response properties and spatiotemporal saccade statistics across different mammals in new and previously published data, and measured visual coverage across time in these animals.

## Materials and Methods

### Adaptation model

For the model, we chose parameters that best matched the average response dynamics (decaying to 30% of the initial response) to brief sequential presentations reported for adaptation experiments in macaques and mice (Nelson, 1991; Priebe et al., 2002; Motter, 2006; Patterson et al., 2013; Jin et al., 2019; Jin and Glickfeld, 2020). The decay time constant $\tau_{\mathrm{decay}}$for all simulations was 60 ms and the recovery time constant $\tau_{\mathrm{recovery}}$ varied between 100 and 900 ms. The initial nonadapted firing rate was 50 spikes/s, and the constant *k* for the divisive gain function was 30.

### Human participants

Five participants of the Institut des Neurosciences de la Timone (22–29 years old, 2 females) took part in the free viewing tasks. Experiments were approved by the Ethics Committee of Aix-Marseille University and conducted in accordance with the Declaration of Helsinki. All participants gave written informed consent.

### Mice

Experiments and procedures were performed on eight C57bl6 adult female and male mice (RRID: JAX: 00664), one female and two male PV-Cre mice (Scholl et al., 2015), two female PV-Cre;Ai14 mice (Scholl et al., 2015), and two female and four male PV-Cre;ChR2 mice (Choi and Priebe, 2020; all from the C57bl6 line of mice). Mice were group-housed and maintained on a 12 h light/dark cycle under standard housing conditions. All procedures and care were performed in accordance with the guidelines of the Institutional Animal Care and Use Committees at the University of Texas at Austin.

### Marmosets

Seven marmosets (1.5–4 years, 2 females) were obtained from TxBiomed and bred in-house and were group-housed. Food and water were provided *ad libitum*. All procedures and care were performed in accordance with the guidelines of the Institutional Animal Care and Use Committees at the University of Texas at Austin.

### Human saccades

For free viewing experiments, participants sat in a quiet and dimly illuminated room, with their head positioned on a chin and forehead rest. The experiments were controlled by a HP Z420 (Hewlett-Packard) computer equipped with an Nvidia Quadro 600 video card (Nvidia). Binocular eye position was recorded using an EyeLink 1000 tower mount (SR Research) at a sampling rate of 2 kHz (1 kHz per eye). The experimental software controlling the display as well as the eye tracking was implemented in Matlab (MathWorks), using the Psychophysics (Brainard, 1997; Pelli, 1997) and EyeLink toolboxes (Cornelissen et al., 2002). Stimuli were presented at a viewing distance of 60 cm on a 32 inch Display++ LCD monitor (Cambridge Research Systems) with a spatial resolution of 1,920 × 1,080 pixels and a vertical refresh rate of 120 Hz. Participants’ gaze position was calibrated with a 13-point custom calibration sequence with sequences validated at the beginning of an experimental session as well as when necessary.

Participants took part in free viewing tasks composed each of 40 runs of eight trials each. These runs were completed in four experimental sessions (on different days) of ∼60 min each (including breaks). Participants were instructed to freely explore a set of 80 images displayed at the center of a gray background screen. These pictures were presented on each trial for a duration of 30 s and separated each by two seconds of blank gray screen. Pictures were selected from the natural images from the McGill Calibrated Color Image Database (Olmos and Kingdom, 2004). For the first 20 runs, participants randomly explored the screen with either the 80 full-sized selected images covering 40 × 32 dva of the visual field or the same 80 images center-cropped covering 20 × 16 dva of the visual field. For the next 20 runs, each participant explored 40 images selected randomly from the same set of 80 images, but with their luminance contrast scaled down uniformly at either 5 or 10% of the original contrast. These images covered 40 × 32 dva of the visual field during the first 10 runs and 20 × 16 dva of the visual field in the last 10 runs. Data from images of both sizes were combined for contrast-related results.

For all experiments, saccades were detected as previously described for marmosets with a velocity threshold (Samonds et al., 2018).

### Marmoset saccades

Marmoset eye position was tracked as previously described using an EyeLink 1000 (SR Research) camera to capture binocular eye position at sampling rate of 1 kHz (500 Hz for each eye; Samonds et al., 2018; Samonds et al., 2019), and saccades were detected with a velocity threshold (Samonds et al., 2018). Twenty-four small (20 × 16 dva) and 24 large (40 × 32 dva) images from the McGill Calibrated Color Image Database (Olmos and Kingdom, 2004) were presented per session on a CRT monitor (85 Hz refresh rate) 50 cm away randomly interleaved them with unrelated 1 s fixation trials using Maestro software suite (https://sites.google.com/a/srscicomp.com/maestro/). In separate sessions, the images were presented with their luminance contrast scaled down uniformly at either 5 or 10% of the original contrast. Data from images of both sizes were combined for contrast-related results.

Marmosets had higher saccade rates than expected with respect to their temporal frequency relationship to the other animals. Previously, we found that marmosets have smaller saccade sizes than we predicted based on their receptive field sizes (Samonds et al., 2018). We attributed this mismatch to marmosets normally using saccadic head movements to make rapid changes in gaze (Mitchell et al., 2014). To probe this idea further, head and eye position were measured in marmosets that were not head restrained using DeepLabCut (Mathis et al., 2018) to track pupils and the center of the upper forehead in 400 × 400 pixel images collected at 60 frames per second (Movie 1). Calibration for these results was done on head-restrained marmosets using the fixation methods described previously (Mitchell et al., 2014, 2015; Movie 2). Saccade statistics based on DeepLabCut of posted marmosets were not distinguishable from those measured with the EyeLink system. For analysis of unposted marmosets, we only looked at continuous segments when the gaze was toward the monitor (within ±50 dva from the monitor center). Note that the colored circles in the videos are tracking the center of the black pupils and not the white corneal reflections.

**Movie 1.** Example of a marmoset freely viewing natural images. Dots are DeepLabCut markers for tracking pupil and head position. [View online]

**Movie 2.** Example of marmoset eye movements when viewing natural images while head-posted using the same camera and tracking setup as used in Movie 1. The bottom two traces show left and right eye horizontal pupil positions over frames. Circles mark the onset time for detected saccades. [View online]

Marmosets clearly used combined head and eye movements for nearly all changes in gaze, which always produced larger gaze changes than with the eyes alone. We did not observe obvious differences in the gaze change rates. Interestingly, marmosets also cock their heads frequently and rapidly, especially when presented with novel objects (Menzel and Menzel, 1980). This might increase their ability to decorrelate orientation-tuned receptive field responses and allow them to sample visual information at a faster rate than with saccades alone.

For comparison with our data, two open circles (median size and rate for each subject) reformatted from a previous publication were added to our plot. The two marmosets were viewing 44 × 34 dva images displayed for 20 s of natural images including humans, macaques, and marmosets (Mitchell et al., 2014).

### Mouse saccades

For image size experiments, saccade statistics are from four C57bl6 and two PV-Cre;Ai14 female mice from a previously reported study (Samonds et al., 2018). These mice were trained for a binocular discrimination task (Samonds et al., 2019) and 8–12 small (40 × 32 dva) and 8–12 large (80 × 64 dva) images/per session were presented before or after 2–3 training sessions. Images from the McGill Calibrated Color Image Database (Olmos and Kingdom, 2004) were presented onto a screen 22 cm away with a DLP LED projector. Binocular eye movements were tracked using custom software to analyze 250 × 250 pixel images collected at 20–30 frames per second with cameras aligned with the orbital axis of each eye. Since mice have a much higher contrast threshold and saturation point than primates, for image contrast experiments, we had them view natural images scaled down to 25 and 50% of the original contrast. This increase for mice compared with primates roughly corresponds to the ratio of increase in contrast thresholds for mice compared with humans (Umino et al., 2018). For three male and one female C57bl6 mice, images were scaled to modulate at a ratio of 0.25 or 0.5 of their original contrast modulation. Experiments were run in the same manner as the previous study except four images each of 25, 50, and 100% contrast were randomly interleaved for two sessions. Mice were first trained to discriminate black from white or crossed versus uncrossed disparities from dynamic random dot stimuli by licking left or right. Natural images were shown before or after a training session when mice reached consistent performance above 75% correct for any task. Eyes were tracked in the same manner as previously described except that DeepLabCut (Mathis et al., 2018) was used to track pupil position. Saccades were detected as previously described with a velocity threshold and comparing detection for both eyes (Samonds et al., 2018).

For monocular deprivation (MD) experiments (Scholl et al., 2017), one eye in each mouse (two female and two male PV-Cre;ChR2) was sutured closed at P24 under anesthesia and sutures were removed at P33. Three age-matched PV-Cre mice (one female and two males) were used as controls. Acuity was assessed and saccades for all mice viewing natural images were measured at P40 (Samonds et al., 2018).

### Macaque saccades

Macaque data were reformatted from figures from a previous publication (median size and rate for each subject), and the two macaques were viewing 44 × 34 dva images displayed for 20 s of natural images including humans, macaques, and marmosets (Mitchell et al., 2014). The median and 25th and 75th percentile was also extracted from the aggregate distribution of both subjects.

### Cat saccades

Cat saccade size statistics (median, 25th percentile quartile, and 75th percentile quartile of reported sizes) were reformatted from figures from previous publications where the cats were either free viewing in the dark (Lee and Malpeli, 1998) or viewing a 21 min natural video of unknown size (Kording et al., 2001). Cat saccade rate statistics (median, 25th percentile quartile, and 75th percentile quartile of intersaccadic intervals) were extracted from horizontal and vertical traces of unpublished data of a head-fixed cat watching a 30 s video of animals at the zoo (unknown size, but smaller than the 60 × 40 dva screen) provided by Theodore Weyand at Louisiana State University School of Medicine. Saccades were detected as previously described for mice and marmosets with a velocity threshold. Dr. Weyand observed that cats made saccades more often for the movie compared with dots or a blank screen (unpublished observations).

### Receptive field size and temporal frequency data

All receptive field size distributions were extrapolated from data from previously published studies that measured the areas or widths of the minimum response field from electrophysiological recordings for cats, marmosets, and macaques, and GCaMP fluorescence thresholded at half the maximum response for mice. Receptive field (RF) size distributions for cats (Albus, 1975; Wilson and Sherman, 1976; Tusa et al., 1978), marmosets (Chaplin et al., 2013), and macaques (Van Essen et al., 1984; Chaplin et al., 2013) were generated by combining receptive field size versus eccentricity data fits with cortical magnification data fits, and for mice, we used the reported distribution (Roth et al., 2016). For more details, see Samonds et al. (2018).

All preferred temporal frequency distributions were aggregated from previously published electrophysiology studies that were based on peak responses in tuning curves generated from average spike rates measured from single neurons in anesthetized animals. For all studies, responses for each temporal frequency were measured using drifting sinusoid gratings centered on the receptive field of single neurons using gratings with the preferred orientation and spatial frequency of the neuron. All spikes were measured extracellularly for single units. All studies used single insulated tungsten electrodes, except a silicon microprobe with 16 recording sites was used to record spikes in mice for one study (Niell and Stryker, 2008). Only summary statistics from the microprobe study were used to validate our aggregate statistics from other studies. For macaques, peak temporal frequencies for 130 single neurons from Foster et al. (1985) were combined with fitted peak temporal frequencies for 75 single neurons from Hawken et al. (1996) to generate an aggregate distribution of peak temporal frequencies. This distribution was consistent with summary statistics reported in additional macaque studies using similar methods (Webb et al., 2005; Priebe et al., 2006; Van den Bergh et al., 2010). For cats, peak temporal frequencies for 36 single neurons from Ikeda and Wright (1975) were combined with fitted peak temporal frequencies for 72 single neurons from Allison et al. (2001) to generate an aggregate distribution of peak temporal frequencies. For mice, fitted peak temporal frequencies for 192 single neurons from Gao et al. (2010) were combined with fitted peak temporal frequencies for 69 single neurons from Van den Bergh et al. (2010) to generate an aggregate distribution of peak temporal frequencies. This distribution was consistent with summary statistics reported in an additional mouse study using similar methods (Niell and Stryker, 2008). From the complete aggregate distributions for each animal, we measured the median and 25th and 75th quartiles.

### Cortical density

Cortical density per dva was estimated by taking the total number of V1 neurons and computing what the total 2D representation of neurons would be based on the surface area and depth of V1 and then dividing that by the total visual area in degrees. We estimated total numbers of neurons using the volume of V1 in mm^3^ times a density of 150,000 neurons/mm^3^ because this density has been a relatively consistent value reported in V1 for chimpanzees (Miller et al., 2014), macaques (O’Kusky and Colonnier, 1982), marmosets (Atapour et al., 2019), and mice (Keller et al., 2018). For humans, we used 1,470 mm^2^ for surface area (Dougherty et al., 2003) and 2.5 mm for depth (DeFelipe, 2011) to get 211 neurons/dva^2^. For marmosets, we used 200 mm^2^ for surface area (Solomon and Rosa, 2014) and 1.1 mm for depth (Balaram and Kaas, 2014) to get 28.7 neurons/dva^2^. For mice, we used 3 mm^2^ for surface area (Garrett et al., 2014) and 0.8 mm for depth (DeFelipe, 2011) to get 0.43 neurons/dva^2^.

### Statistics and reproducibility

All statistical tests were nonparametric based on the median, and error bars were based on bootstrap analysis of the median by resampled data 1,000 times, allowing repeats, to produce surrogate datasets of the same size. The 160th and 840th samples were used for the standard error of the median for all results. For data we collected on humans, marmosets, and mice, we present the entire distribution of all subjects grouped or single subject medians as single solid data points, an outline connecting the standard error of the median of grouped data across rate and size distributions, and an outline connecting the 25th and 75th quartiles of grouped data across rate and size distributions. For receptive field, temporal frequency and saccade data published previously by other authors, we present the median of grouped data or medians of single subjects as open circles and a dashed outline connecting the 25th and 75th quartiles of grouped data across *y*- and *x*-axis distributions. For statistical tests, bootstrapped data for one set of observations were used to find the percentile equal to the median of the compared set of observations. If the compared median was completely outside of the bootstrapped dataset, we reported that as *p* < 0.001.

### Data and code availability

The data presented in the figures and the model code used to generate the figures are available at Figshare: https://figshare.com/projects/Mammals_achieve_common_neural_coverage_of_visual_scenes_using_distinct_sampling_behaviors/136912.

## Results

The adaptation properties of cortical neurons are remarkably distinct across mammals. For both mice and macaques, the responses to brief presentations (<0.5 s) within visual receptive fields of the cortex decay within a couple hundred milliseconds, but macaque neurons recover their responsiveness very quickly (within a few hundred milliseconds; Fig. 1*A*,*C*, blue; Muller et al., 1999; Priebe et al., 2002; Motter, 2006; Patterson et al., 2013) while mouse neurons recover much slower (over several seconds; Fig. 1*B*,*C*, red; Jin et al., 2019; Jin and Glickfeld, 2020). Recovery time constants have not been explicitly measured in cat neurons in the cortex, but the available data suggests that they recover with a time constant somewhere in between these two values (Nelson, 1991). Recovery to adaptation for these animals in these studies were measured for repeated presentations of the same stimuli while the animal was fixating or anesthetized. Differences in the recovery time constants measured among studies in the same species for different visual cortical areas or experimental conditions were negligible compared with differences across species. Saccadic eye movements that decorrelate input across fixations may decrease the effects of neural adaptation (Samonds et al., 2018), but the adaptation that persists with even large changes to visual input nonetheless requires time for recovery (Patterson et al., 2013). Macaque neurons will still recover much faster than mouse neurons even with large enough saccades.

To quantify how different saccade rates influence adaptation, we constructed a conceptual model in which the dynamics of adaptation could be incorporated into the theoretical framework we previously used to explain the relationship between neural adaptation and saccade size (Samonds et al., 2018). Responses (*r_m_*) of a population of *M *= 500 neurons to *N *= 500 stimuli (*s_n_*) following sequential saccades were multiplied by a divisive change in gain dependent on the response to the previous stimulus (*s_n_*_−1_):

$$r_{m\;\mathrm{adapted}}(s_{n})=\;\frac{k}{k+r_{m}(s_{n-1})}r_{m}(s_{n}).$$ (1)

Parameters of this function (*k* and average responses) were chosen to approximately match observed adaptive changes in gain in V1 of macaques, mice, and cats, where the average gain never reaches zero (on average 30% of peak responses) for even the fastest repeat presentations (see Extended Data Fig. 1-1 and Materials and Methods for details; Nelson, 1991; Patterson et al., 2013; Jin et al., 2019; Jin and Glickfeld, 2020). We varied the time between stimuli to represent different saccade rates and model responses decayed over time (*t*) as an exponential decay function with a fixed time constant $\tau_{\mathrm{decay}}$. The divisive gain based on the initial response after each saccade recovered over time based on a second exponential decay function:

$$r_{m\mathrm{adapted}}(t\;s_{n})=\;\frac{k}{k+e^{\left((-t)/\tau_{\mathrm{recovery}}\right)}\;r_{m}(1\;s_{n-1})}r_{m}(1\;s_{n})\;e^{\left((-t)/\tau_{\mathrm{decay}}\right)}$$ (2)

We varied the time constant $\left(\tau_{\mathrm{recovery}}\right)$ of this function to cover a range that includes experimentally observed adaptation recovery time constants to brief presentations of stimuli in V1 of macaques and mice (Patterson et al., 2013; Jin et al., 2019; Jin and Glickfeld, 2020). To mimic the sensory effects of saccades, we repeated this analysis but changed the stimulus. For some conditions, the stimulus changed randomly, thus providing a decorrelated input mimicking large saccades (Fig. 1*D*, solid lines). For other conditions, the stimulus did not change (no decorrelation), mimicking small saccades relative to receptive field sizes (Fig. 1*D*, dashed lines). As saccade rate increases, neurons have less time to recover from adaptation and the overall responses decline (Fig. 1*D*). For both animals, large saccades reduced adaptation compared with small saccades yielding comparatively higher responses for faster saccade rates, reflecting a linkage between saccade size and rate (Fig. 1*D*, solid vs dashed lines). To illustrate how the recovery time would relate to saccade rate, we plotted the saccade rate that would still maintain 75% of responsiveness with decorrelation (large saccades) against the recovery time constant $\tau_{\mathrm{recovery}}$ (Fig. 1*E*). This simple model (Fig. 1*E*, black) predicts a dramatic difference in saccade rate that depends on the adaptive dynamics in visual neurons and captures the large differences in saccade rates observed between macaques (Fig. 1*E*, blue data from Patterson et al., 2013; Mitchell et al., 2014) and mice (Fig. 1*E*, red data from Samonds et al., 2018; Jin and Glickfeld, 2020) based on their V1 recovery time constants.

Our model suggests that there should be a functional link between the spatiotemporal dynamics of the visual and motor systems. To characterize the spatiotemporal tradeoffs made by these two systems, we first recorded and compared the saccade rates and sizes when presenting natural scenes to three different species known to have different visual system properties: humans, marmosets, and mice (Fig. 1*F*). As demonstrated previously (Samonds et al., 2018), there are clear differences in saccade sizes between these species, with all animals exhibiting a skewed distribution in saccade amplitude (Extended Data Fig. 1-2*B*). Marmosets have the smallest saccades and mice have the largest ones. For all animals, saccade sizes were slightly larger for larger images (see also von Wartburg et al., 2007; Otero-Millan et al., 2013; Extended Data Fig. 1-2*B*; bootstrapped, *p* < 0.001 for all comparisons). There are also clear differences in saccade rates between species (Fig. 1*F*). Marmosets have the highest saccade rates and mice have the lowest saccade rates. For all animals, saccade rates were slightly higher for larger images (see also Otero-Millan et al., 2013; Extended Data Fig. 1-2*C*; bootstrapped, *p* < 0.001 for all comparisons). Saccade behavior is similar between animals with only a shift in rates and sizes. Including previously reported data from other animals (open circles for marmosets, macaques, and cats) reveals an inverse relationship between saccade rate and saccade size across distinct mammalian species (Fig. 1*F*). The saccade size and rate statistics reported in several publications are remarkably similar to our data when the authors employed similar “free viewing” approaches and image size and duration are considered (Tatler et al., 2006; Jansen et al., 2009; Nikolaev et al., 2013; Otero-Millan et al., 2013; Loh et al., 2022). Humans and all animals were passively viewing natural images of the same or similar size as humans and were prevented from making head movements. One distinction was that mice were allowed to freely run, but their data were similar whether they were running or stationary (except a lower rate when stationary; Samonds et al., 2018).

The decorrelation hypothesis predicts that saccade size differences between species are attributed to the properties of their respective visual systems (Samonds et al., 2018). Human and macaque receptive fields are small relative to mouse receptive fields, whereas marmoset and cat receptive fields lie between these sizes. Consequently, to achieve decorrelation and sample new information, mice need much larger saccades in order to move their larger receptive fields as compared with humans and the other species. Our model also suggests that saccade rates will be matched to differences in adaptation recovery time constants (Fig. 1*E*). Since adaptation time constants in individual animals can vary depending on experimental conditions and data based on similar conditions is fairly limited across species, we rather considered preferred temporal frequency distributions across several species to provide a more comprehensive view of the relationship between spatial and temporal properties of their visual system neurons. While temporal frequency tuning curves are a steady-state measurement using a different experimental paradigm than the transient measurements of recovery time constants of our model, psychophysically the time course of adaptation is directly related to the temporal frequency of drifting gratings (Lorenceau, 1987). Consistent with psychophysics, neurophysiological measurements have linked transient and steady-state response properties with the temporal components of visual drive (Baker, 1988; Nelson, 1991; Lisberger and Movshon, 1999). Indeed, temporal frequency tuning has been measured in several animals under very similar experimental conditions (e.g., spike rate responses to sinusoidal luminance gratings; see Materials and Methods) in different laboratories (Ikeda and Wright, 1975; Foster et al., 1985; Hawken et al., 1996; Allison et al., 2001; Webb et al., 2005; Priebe et al., 2006; Niell and Stryker, 2008; Gao et al., 2010; Van den Bergh et al., 2010; Yu et al., 2010; Durand et al., 2016). Similar to the saccade statistics, there is an inverse relationship between preferred temporal frequency and receptive field size across these animals (Fig. 1*G*). Both the oculomotor and visual systems share spatiotemporal tradeoffs across species where those with smaller receptive fields can make smaller saccades but require more frequent sampling and faster visual processing. Indeed, we find that low luminance contrast of natural images, which it is known to shift temporal frequency tuning lower (Alitto and Usrey, 2004; Priebe et al., 2006; Camillo et al., 2020), is associated with a corresponding reduction in saccade rate (Extended Data Fig. 1-3).

The relationship we uncover between saccade size and rate may result from differences between species other than receptive field sizes. To test whether changes in receptive field size are linked to saccade rate, we reduced the spatial acuity of mice using MD during the critical period (age 24–32 days). This manipulation shifted their saccades to larger sizes (Fig. 1*H*, top; Samonds et al., 2018) and significantly reduced their saccade rates compared with control mice (Fig. 1*H*, bottom; bootstrapped, *p* < 0.001). This causal manipulation demonstrates that the inverse relationship found between saccade size and rate is related to visual functional properties such as acuity. One of these properties is presumably receptive field sizes and although we did not measure receptive field sizes directly in these mice, previous studies have found increases in receptive field sizes in cats due to MD (Swindale and Mitchell, 1994), and MD disrupts spatial frequency tuning and the match of orientation selectivity across eyes (Wang et al., 2010; Scholl et al., 2017; Brown and McGee, 2023). This mismatch in orientation selectivity between the eyes could contribute to reduced acuity that are not incorporated into our simple receptive field model for predicting saccade sizes (Samonds et al., 2018). A similar change in saccade size and rate is observed in humans on a coarse scale with substantial reduced acuity due to macular degeneration or foveal occlusion (Kwon et al., 2013; Seiple et al., 2013) and on a finer scale in subjects with myopia and amblyopia (Chen et al., 2018; Tang et al., 2019).

Our modeling and empirical data in different species show that visual and motor systems share similar spatiotemporal tradeoffs to sample visual information. We reasoned that such a tradeoff is constrained by a common computational goal: achieving equivalent novel (decorrelated) spatiotemporal coverage of their visual environment. The mouse with both large V1 receptive fields and eye movements would need to make eye movements less frequently compared with the human with both small receptive fields and eye movements in order to achieve similar updated coverage over time. There appear to be notable exceptions to the general inverse relationship between saccade rate and size across species though. First, humans appear to have lower saccade rates than macaques although they have slightly smaller saccade sizes (Fig. 1*F*; see also Berg et al., 2009). Second, if we assume that marmosets make larger saccades with their heads (Mitchell et al., 2014) with no decrease in saccade rate (Movies 1–2 and Extended Data Figs. 1-4 and 1-5), they would have higher saccade rates than macaques with larger saccade sizes. This suggests that saccade rates might also depend on species-specific factors other than saccade sizes and receptive field sizes.

To assess coverage over time quantitatively and better understand why different species have different saccade rates, we applied a circular mask equal to the median V1 receptive field size for each animal to every fixation point (Fig. 2*A*, green circles). We then added up all of those masks (each equal to one for that area) across space and time for all images to generate cumulative spatial maps. Lastly, we divided those maps by the total of the intersaccadic interval times to get a percent coverage per second (Fig. 2*A*, right). Because coverage is based on area, the mouse achieves much more coverage over time compared with marmosets and humans even with their slower saccade rates (Fig. 2*B*).

10.1523/ENEURO.0287-23.2023.f2-1 Extended Data Figure 2-1 **Saccade size and rate decrease with decreasing image size and novelty** **A**, Saccade rate versus size distributions for small and large images for humans, marmosets, and mice (same data as Fig. 1f and Supplemental Fig. 2). Lightest colors represent data for n = 7 marmosets performing fixation training (n = 8,738 saccades) and n = 6 mice discriminating DRDS disparity (n = 2,016 saccades). Each solid point represents the medians of a single subject. The large outline represents 25th and 75th percentiles and the small cross represents the standard error of the median for the distribution of all subjects. Due to the large number of samples, the standard error of the median outlines are smaller than even the data points. For all species, the saccade size and rate were substantially and significantly smaller for these tasks compared to saccades generated when viewing natural images (bootstrapped, p < 0.001 for all comparisons). **B**, Image size is not the only dimension that factors into the amount of sampled visual information over time. Visual information also becomes redundant and less informative over time, if there are no changes or updates in visual information, as for instance with static images. We measured changes in saccade rate and size over the entire 30 s presentations for humans, marmosets, and mice. Saccade rate versus size over time for n = 5 humans (n = 10,172; n = 8,949; and n = 7,500 saccades), n = 7 marmosets (n = 6,070; n = 5,284; and n = 4,173 saccades), and n = 6 mice (n = 947 and n = 408 saccades) viewing large images. For humans and marmosets, saccade rates and sizes quickly decreased over time (within the first 5 s of viewing an image; bootstrapped, p < 0.001 for all comparisons). This decrease was quicker for animals that saccade at faster rates. Marmosets had a quicker decrease in rates and sizes compared to humans (bootstrapped, 14.7 vs 8.9% and 9.6 vs 5.4% decrease in the first 5 s, p < 0.001 and p = 0.008, respectively) and there was only a noticeable decrease in saccade rates and sizes for mice after viewing an image for 20 s (bootstrapped, p = 0.06 and p = 0.003, respectively). Download Figure 2-1, TIF file.

10.1523/ENEURO.0287-23.2023.f2-2 Extended Data Figure 2-2 **Saccade and fixation dynamics whiten natural inputs to V1** Plots of spatial frequency distributions of model V1 receptive fields for mice (solid red) and macaques (solid blue) used to predict saccade sizes based on decorrelation of responses2 compared to the attenuation of spatial frequencies relative to natural images caused by >7-degree saccades (dashed red) and 3-4-degree saccades (dashed blue). We estimated attenuation by dividing the spectral data from Fig. 4C by the spectral density of natural images in Mostofi et al. (2020). Larger saccades and lower temporal frequencies shift the attenuation curve to lower spatial frequencies, while smaller saccades and higher temporal frequencies shift the attenuation to higher spatial frequencies. Download Figure 2-2, TIF file.

We postulated that the main bottleneck constraining coverage over time is the receptive field size and density of the first cortical stage, the primary visual cortex. The animals we are examining have very different densities of V1 cortical neurons per degree of visual angle (dva) and therefore differences in densities of feature preferences (Jang et al., 2020; Fig. 2*C*). Humans have many more neurons for each receptive field location representing many more different visual features than mice, and mathematically, larger networks can provide greater decorrelation (Fasoli and Panzeri, 2019). We can take our original coverage maps over time (Fig. 2*B*) and multiply by the two-dimensional (2D) V1 neuronal density for each animal (see Materials and Methods for details) to compare receptive field cortical coverage over time between species (Fig. 2*D*). Because marmosets often use head movements for gaze changes and their eye positions, unlike humans, appear to be restricted to mostly ±10 dva (Fig. 2*B*, center; see also Movies 1–2 and Extended Data Figs. 1-4 and 1-5 and Mitchell et al., 2014), we observe comparable coverage for smaller 20 dva images between humans and marmosets (Fig. 2*D*, center, light gray vs light cyan). If marmosets had receptive field sizes equal to macaques, their coverage would be reduced by nearly 90%, and if marmosets had saccade rates equal to humans, their coverage would be reduced by nearly 30%. Mice also use head movements for changes in gaze (Meyer et al., 2020; Michaiel et al., 2020; Zahler et al., 2021), but their eye positions extend out to at least cover ±20 dva (Fig. 2*B*, right) so we observe comparable coverage for 40 dva images between humans and mice (Fig. 2*D*, right, dark gray vs red). These comparisons suggest that neuronal densities counteract receptive field area differences and saccades for these mammals are decorrelating similar numbers of neurons over time despite having very different saccade rates, saccade sizes, and receptive field sizes. With this sampling strategy, differences in saccade rates and sizes between animals are explained by corresponding differences in receptive field sizes and cortical densities with respect to visual space. If saccade rates and sizes are calibrated to achieve a certain level of visual coverage, this would also explain why saccade rates and sizes change with image size and decrease over viewing time (Extended Data Figs. 1-2 and 2-1; see also von Wartburg et al., 2007; Otero-Millan et al., 2013; Samonds et al., 2018). As our model predicts, saccade rates are always higher in the condition where it is easier to achieve decorrelation. The oculomotor system indeed seems to favor larger versus smaller saccades, since it is easier and faster to make larger saccades (Harwood et al., 2008; De Vries et al., 2016; Poletti et al., 2020).

## Discussion

We propose that gaze changes efficiently sample visual scenes based on natural statistics, neural receptive fields, and adaptation, but this hypothesis makes some important assumptions. First, for the purposes of predicting saccade sizes, we assumed that the receptive field is static and only examined image differences across sequential fixations (Samonds et al., 2018). In fact, saccades have complex dynamics over 10’s of milliseconds, and the eyes drift and oscillate continuously over 100’s of milliseconds or seconds of fixation. These fast and slow oculomotor dynamics effectively whiten the visual inputs (Kuang et al., 2012; Segal et al., 2015; Mostofi et al., 2020). This whitening is typically outside of the frequency spectrum of the V1 response preferences that we used to predict saccade sizes (Extended Data Fig. 2-2). Indeed, V1 responses following a saccade are very similar to those evoked by sequentially flashed stimuli (Parker et al., 2022). Nonetheless, saccades can produce an intra-saccadic flow of information that is sensed (Castet and Masson, 2000; Castet et al., 2002), can boost post-saccadic visual motion processing (Miles et al., 1986), and have functional consequences for spatial vision (Schweitzer and Rolfs, 2020, 2021). Additionally, our simplified experiments and conceptual model only systematically changed the sensory stimulus. Motor signals arising from the saccade itself, such as a corollary discharge, might also influence V1 responses in complex ways (Super et al., 2004; Wurtz, 2008; McFarland et al., 2015). Second, we assumed that the primary visual cortex was the main processing bottleneck that constrains saccade sizes and rates. This choice was partly made because of the large number of comparable data available across multiple species. This assumption does not rule out that differences in receptive fields found earlier, later, or subcortically in the visual system between species contribute to their oculomotor differences. Future work shall include additional processing that occurs during both saccades and fixation and how they influence both cortical and subcortical responses. Another possibility that we did not address in this work is that the oculomotor spatial and temporal species-specific properties have actually contributed to shape the properties of the visual system rather than the oculomotor behavior being the consequence of the visual system characteristics (Rolfs and Schweitzer, 2022).

Yarbus illustrated that humans move their eyes deliberately toward objects and features of interest and the overall pattern of those movements can heavily depend on the task (Yarbus, 1967). Decades of research support that the primary purpose of saccadic eye movements is to move an area of interest into the high-spatial acuity portion of the visual field (Yarbus, 1967; Koch and Ullman, 1985; Hayhoe and Ballard, 2005; Najemnik and Geisler, 2005; Gegenfurtner, 2016). Even animals with no fovea, such as mice, use gaze changes to move objects of interest into the central portion of their upper visual field (Michaiel et al., 2020; Johnson et al., 2021) where binocularity and spatial features are represented better (Samonds et al., 2019; van Beest et al., 2021; Tan et al., 2022). Our hypothesis is complementary to foveation and does not attempt to predict saccade targets instantaneously. We use decorrelation as a measurement to quantify a statistical sampling strategy for natural scenes over a long time scale of several fixations to update an internal visual representation. It is important to point out that this sampling strategy can depend on and adapt to environmental and task demands as well. We see this when varying image contrast and size (Extended Data Figs. 1-3 and 2-1; see also von Wartburg et al., 2007; Otero-Millan et al., 2013), but previous studies have also shown predictable changes in coverage (size/rate) statistics using images with biased spatial frequency content (Groner et al., 2008), tasks with high acuity demands (Intoy and Rucci, 2020), or tasks with high cognitive demands (Tatler et al., 2006; Loh et al., 2022). Previous work examining transsaccadic integration also shows that information from past fixations influence perception during the current fixation. If that past information about a particular visual feature was more reliable than the current information, it has a stronger influence on perception (Niemeier et al., 2003; Ganmor et al., 2015; Oostwoud Wijdenes et al., 2015; Wolf and Schutz, 2015). Finally, a higher rate of saccades improves performance in detecting changes within a scene (Henderson and Hollingworth, 2003) highlighting the importance of maintaining sufficient coverage. Overall, our analyses illustrate that the oculomotor and visual systems of multiple mammals coordinate to sample the environment efficiently under a diverse range of processing constraints.

## Synthesis

Reviewing Editor: David Schoppik, New York University Decisions are customarily a result of the Reviewing Editor and the peer reviewers coming together and discussing their recommendations until a consensus is reached. When revisions are invited, a fact-based synthesis statement explaining their decision and outlining what is needed to prepare a revision will be listed below. The following reviewer(s) agreed to reveal their identity: Olivier Marre, J. Patrick Mayo.

1) How are the time constant of adaptation measured?

Fig 1e shows a striking relation between the saccade rate and the recovery time constant, but it is not clear how the latter was measured. Was it learned from data ? Or estimated from previous papers? In this figure, it is also not clear what was the saccade size. The previous panel suggests that the relation will change with the saccade size. It is not clear which size was chosen. If the constants were not learned from data, it is easy to find values that make the two points fall on the line.

2) Since it is not always easy to access the value of the adaptation time constant, the authors used the temporal frequency tuning as a proxy. It is not clear to us if it is so easy to go from one to the other. The temporal frequency tuning assumes a linear model, while the adaptation model assumes a underlying non)linear model. The link between the two should be clarified.

3) The logic of figure 1f-g is that saccade size is determined by receptive field size, which is itself correlated with temporal frequency tuning, itself connected with adaptation time constant and then saccade rate. This is quite an indirect connection which probably explains why the relation between saccade size and saccade rate does not seem straightforward across species. The authors mention the cases where this relation does not seem to hold, but one is left to wonder exactly what is the extent of their claims. Is it really a general law with only a few exceptions ?

4) The experiment on monocular deprivation is very interesting. However, it seems that MD could impact many aspects of the visual system, not just the receptive field size. A more convincing point could be made if the change in saccade size could be predicted by the change in receptive field size. This would imply to measure the latter was experimentally measured by the authors.

5) Figure 2 aims at demonstrating that the different combinations of RF size and saccade amplitude ensure the same coverage. This sounds like an attractive hypothesis, but in practice the main factor seems to be the density of cells, which vary a lot across species. At least one would expect a control where the saccade amplitude distribution is permuted between species. Does this lead to a less uniform coverage across species ?

6) It is difficult for us to accept that large saccades are equated with consecutive presentations of different visual stimuli, while small saccades are equated with presentations of the same stimulus (Lines 303-304; also Line 323.) This seems like an extremely poor way to approximate "large" vs. "small" saccades. Saccades involve at least some level of active, anticipatory stabilizing circuitry via corollary discharge/efference copy and alter neuronal responses at most (every?) levels of the visual system, as well as perception, even before and after the eyes move. The authors' approach is particularly problematic because they go on to assert relationships between "motor" and sensory components (e.g., Line 393), when all that was done experimentally was change the sensory stimulus.

7) The authors use a variety of macaque cortical responses interchangeably (V1 vs MT; anesthetized responses vs awake). On the one hand, we can imagine these differences playing a major role in the findings (e.g., differences between monosynaptically connected brain regions; IT and PFC in Verhoef...Vogels 2008). On the other hand, we're aware there is relative consistent adaptation across even cortical and subcortical brain regions (FEF and SC in Mayo and Sommer 2008). Because of these issues, we think it would be useful if the authors limited themselves to a single brain region (e.g., V1) and behavioral state (awake, behaving) across species. The authors allude to this slipperiness in the Discussion (Lines 456+) and seem to state that intended to limit themselves to primary visual cortex (Lines 450 and 456); yet the Methods state that they used a model that, as far as I can tell, relied on MT data (Priebe et al 2002) and anesthetized V1 responses (Patterson et al. 2013; Line 71). We appreciate that the temporal frequency data required anesthetized responses because of limited data and we found that explicit statement useful (Lines 219-221). Please clarify what brain regions' data were used and why.

Minor points

Line 4: both "several fixations" and "more complete" seem like they could simply be "fixations" and "complete"

Line 278: Regarding the major point above, please state whether these are primary visual cortical neurons or simply any visually responsive neurons (or something else).

Line 301: We don't think "allow" is an appropriate word here. We understand that the authors would like to imply causality, but I don't think the data warrant that.

Line 408: Berg...Munoz (J. Vision) 2009 shows significantly different saccade amplitudes between macaques and humans (their Fig. 4A), in line with the authors' findings.

Figure 1 Legend: References are in number format, presumably from a prior submission. Please fix.

Supplementary Figure 1 (and related text): Particularly for the macaque data, Motter 2006 (J Neurosci) seems very relevant, particularly if you want to address cortical areas beyond V1.

Supplemental Video 2: I would trim off the last ~1 second of the video so that the movie does not stop playing on a frame where the tracking algorithm is completely missing one eye and partially missing the other.
