# Supplementary figures and images for: Mammals Achieve Common Neural Coverage of Visual Scenes Using Distinct Sampling Behaviors
Source: eNeuro. 2024 Feb 6;11(2):ENEURO.0287-23.2023. doi: 10.1523/ENEURO.0287-23.2023 (PMC10860624; doi:10.1523/ENEURO.0287-23.2023)

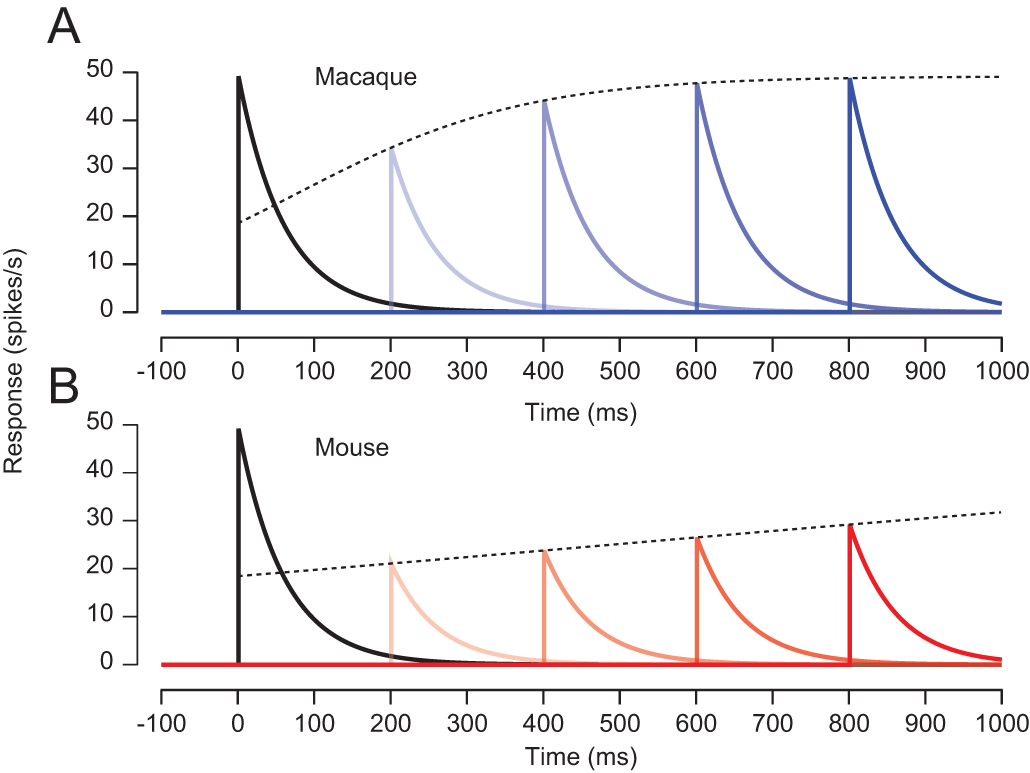

Supplement: Extended Data Figure 1-1 — Example V1 responses modeled with a divisive gain time constant A, Example model macaque V1 responses to two repeated presentations of the same stimulus. Black represents neural activity to the first presentation and different shades of blue depict the neural activity after the second presentation in progressively more delayed conditions. B, Same format as a for mouse model V1 responses. Download Figure 1-1, TIF file. [file eneuro-11-ENEURO.0287-23.2023-s002.tif]

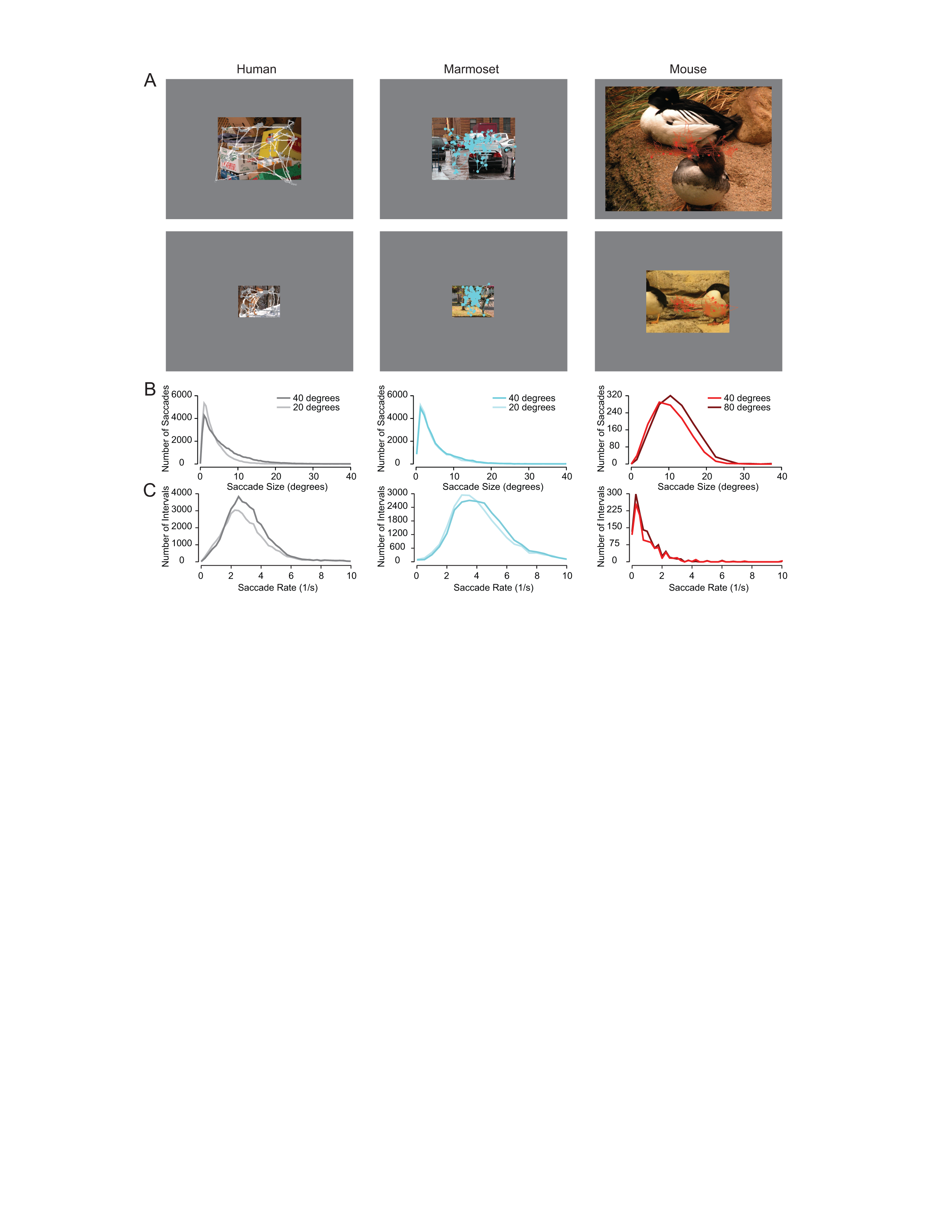

Supplement: Extended Data Figure 1-2 — Saccadic eye movement statistics while viewing natural images A, Eye traces and fixation points (circles) for humans, marmosets and mice viewing an example large (top row) or small (bottom row) image for 30 s. For mice, we plotted fixation points with respect to an assumed projection of binocular photoreceptors by aligning the center of the image with the median eye position, since standard fixation calibration procedures are not possible. B, Histograms of saccade sizes for N = 5 humans each viewing 80 large (n = 49,907 saccades) and small (n = 42,523 saccades) images, N = 7 marmosets each viewing 24 large (n = 25,531 saccades) and small images (n = 24,781 saccades), and N = 6 mice each viewing 8-18 large (n = 1,355 saccades) and small (n = 1,145 saccades) images (Samonds et al. 2018). C, Histograms of saccade rates (1/intersaccadic interval) for the same subjects. Download Figure 1-2, TIF file. [file eneuro-11-ENEURO.0287-23.2023-s003.tif]

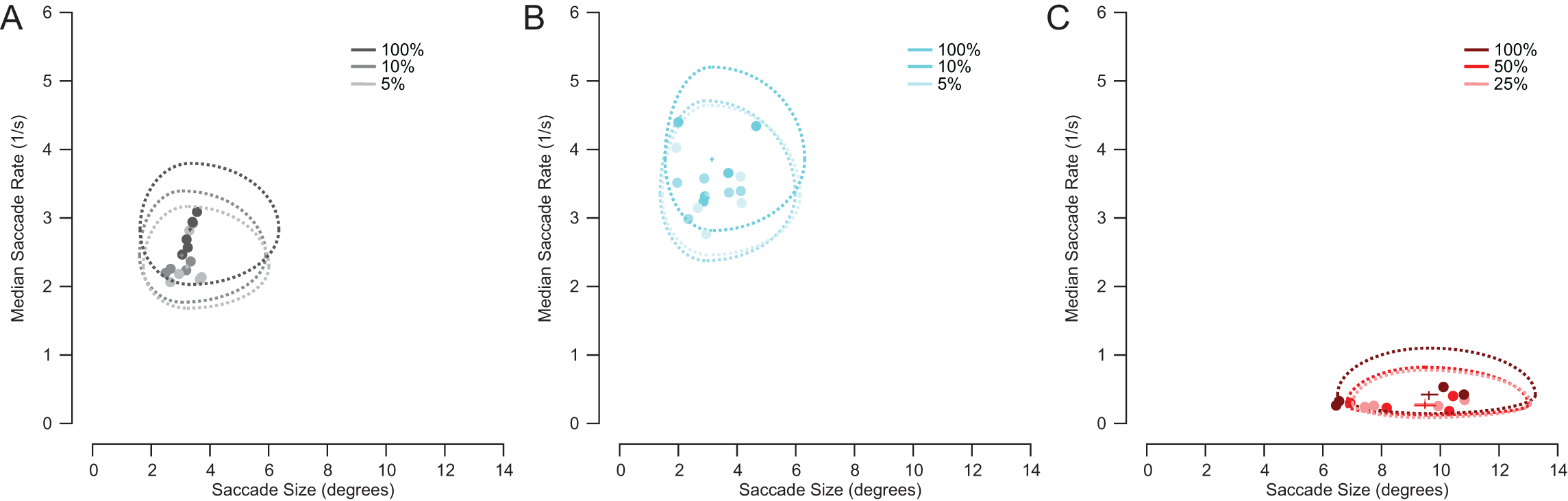

Supplement: Extended Data Figure 1-3 — Saccade rates decrease with decreasing contrast A, Each solid point represents the medians of a single subject. The large outline represents 25th and 75th percentiles and the small outline represents the standard error of the median for the distribution of all subjects. Due to the large number of samples, the standard error of the median outlines are smaller than even the data points. Saccade rate versus size distributions for n = 5 humans for images with progressively reduced contrast (small and large images combined). (100%: n = 92,430 saccades; 10%: n = 42,767 saccades; 5%: n = 41,639 saccades). B, Same data for n = 5 marmosets. (100%: n = 40,474 saccades; 10%: n = 31,927 saccades; 5%: n = 37,294 saccades). C, Same data for n = 4 mice. (100%: n = 472 saccades; 50%: n = 338 saccades; 25%: n = 274 saccades). For all three animals, there was a clear decrease in saccade rate for the images with reduced contrast (from dark to light colors; bootstrapped, 5 and 10 versus 100%, p < 0.001 for all comparisons for humans and marmosets; 25 and 50 versus 100%, p = 0.008 and 0.01, respectively for mice). Download Figure 1-3, TIF file. [file eneuro-11-ENEURO.0287-23.2023-s004.tif]

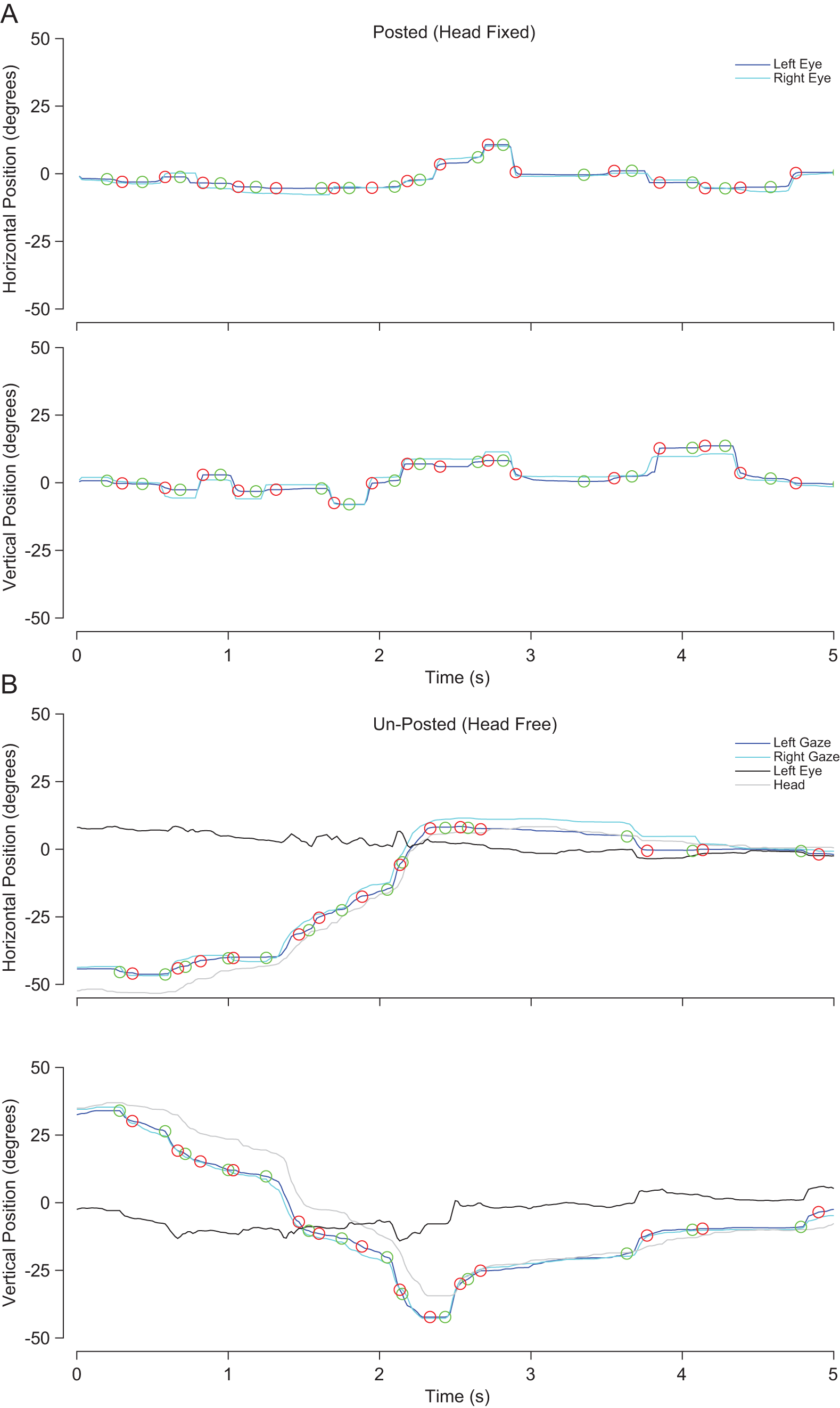

Supplement: Extended Data Figure 1-4 — Gaze position over time for posted and unposted marmosets A, Horizontal and vertical pupil position for both eyes for a head-fixed marmoset. Green circles are saccade onset and red circles are saccade offset. B, Same gaze data for the same marmoset now able to move their head freely unposted (head-free). Forehead position and left eye position relative to the head are included with the gaze traces. Note that the eyes never rotate out more than ±15 degrees and head movements drive the gaze changes. Download Figure 1-4, TIF file. [file eneuro-11-ENEURO.0287-23.2023-s005.tif]

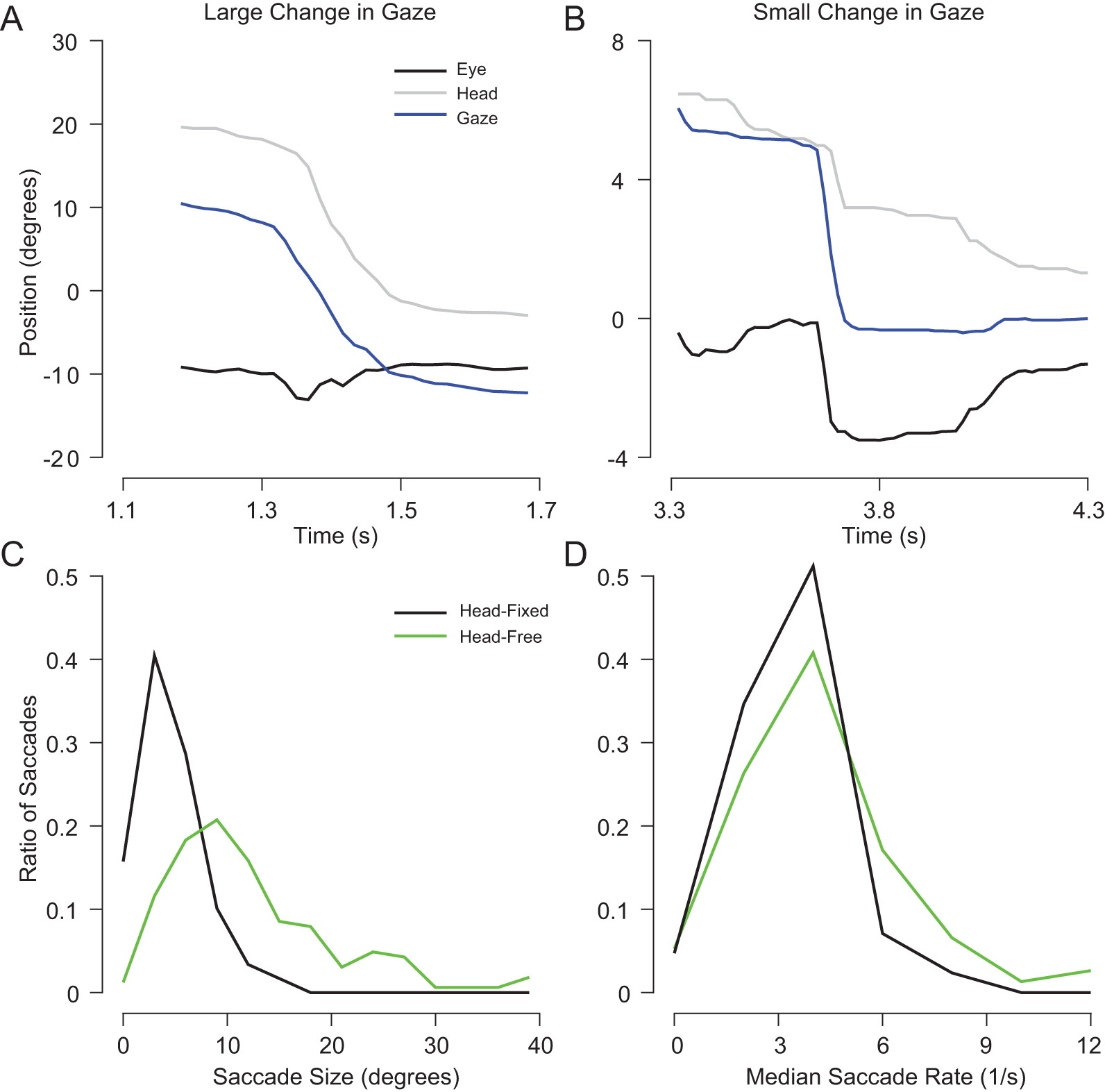

Supplement: Extended Data Figure 1-5 — Contribution of head and eye movements to marmoset gaze changes A, An example of a large change in gaze that is mostly driven by a saccadic head movement. Vestibular ocular reflex (VOR) movements at the beginning and end of the head movement lead to an overall faster gaze changes separated by two stable periods of fixation. B, An example of a small change in gaze. Although most of the movement is the result of the eyes, there is still a significant contributing saccadic head movement and there are similar VOR dynamics as those observed with a large change in gaze. C, Distributions of saccadic gaze change sizes for head fixed and freely moving (head-free) marmosets. D, Distributions of saccadic gaze change rates for head fixed and head-free marmosets. Download Figure 1-5, TIF file. [file eneuro-11-ENEURO.0287-23.2023-s006.tif]

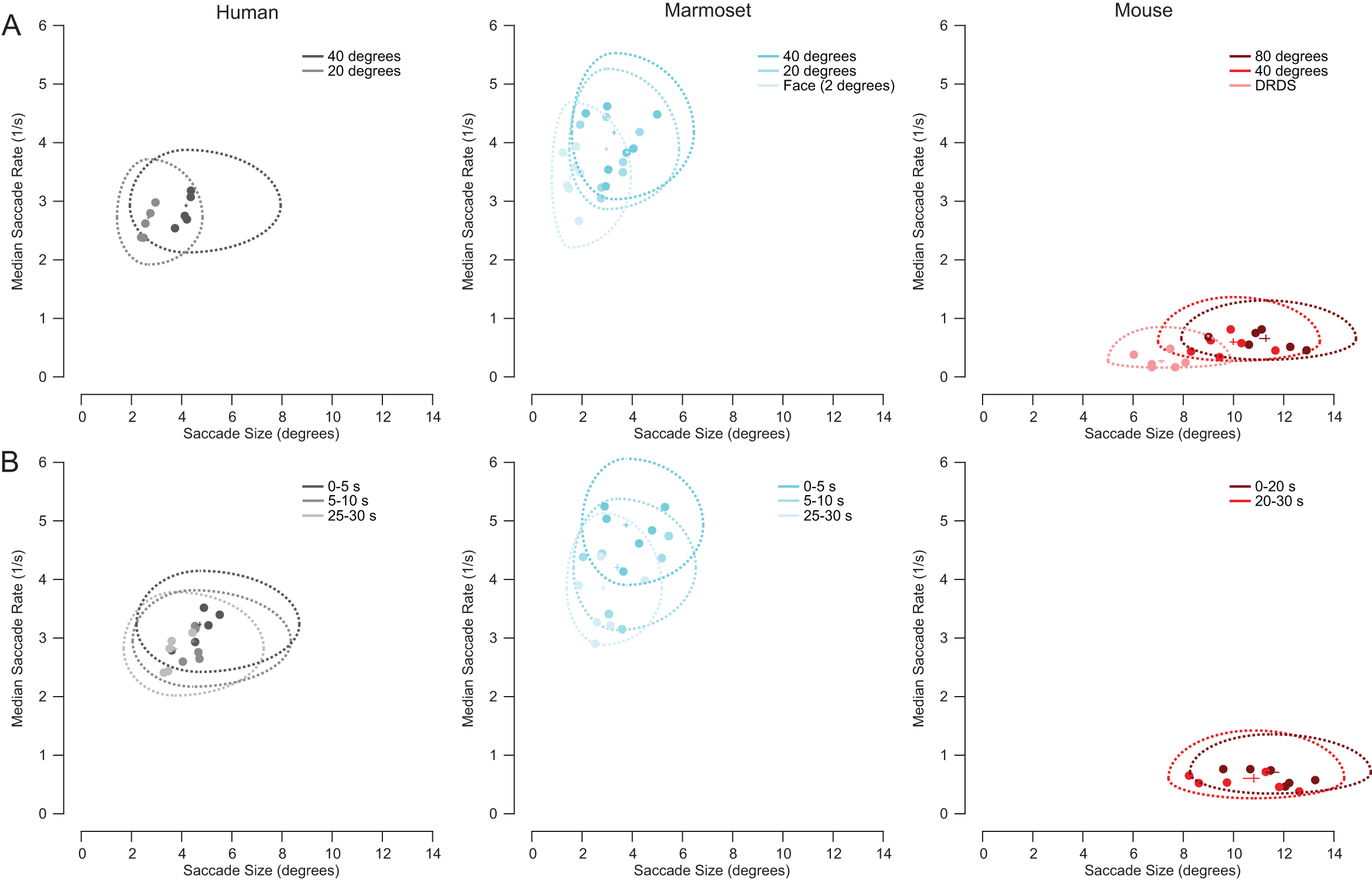

Supplement: Extended Data Figure 2-1 — Saccade size and rate decrease with decreasing image size and novelty A, Saccade rate versus size distributions for small and large images for humans, marmosets, and mice (same data as Fig. 1f and Supplemental Fig. 2). Lightest colors represent data for n = 7 marmosets performing fixation training (n = 8,738 saccades) and n = 6 mice discriminating DRDS disparity (n = 2,016 saccades). Each solid point represents the medians of a single subject. The large outline represents 25th and 75th percentiles and the small cross represents the standard error of the median for the distribution of all subjects. Due to the large number of samples, the standard error of the median outlines are smaller than even the data points. For all species, the saccade size and rate were substantially and significantly smaller for these tasks compared to saccades generated when viewing natural images (bootstrapped, p < 0.001 for all comparisons). B, Image size is not the only dimension that factors into the amount of sampled visual information over time. Visual information also becomes redundant and less informative over time, if there are no changes or updates in visual information, as for instance with static images. We measured changes in saccade rate and size over the entire 30 s presentations for humans, marmosets, and mice. Saccade rate versus size over time for n = 5 humans (n = 10,172; n = 8,949; and n = 7,500 saccades), n = 7 marmosets (n = 6,070; n = 5,284; and n = 4,173 saccades), and n = 6 mice (n = 947 and n = 408 saccades) viewing large images. For humans and marmosets, saccade rates and sizes quickly decreased over time (within the first 5 s of viewing an image; bootstrapped, p < 0.001 for all comparisons). This decrease was quicker for animals that saccade at faster rates. Marmosets had a quicker decrease in rates and sizes compared to humans (bootstrapped, 14.7 vs 8.9% and 9.6 vs 5.4% decrease in the first 5 s, p < 0.001 and p = 0.008, respectively) and there was only a noticea [file eneuro-11-ENEURO.0287-23.2023-s007.tif]

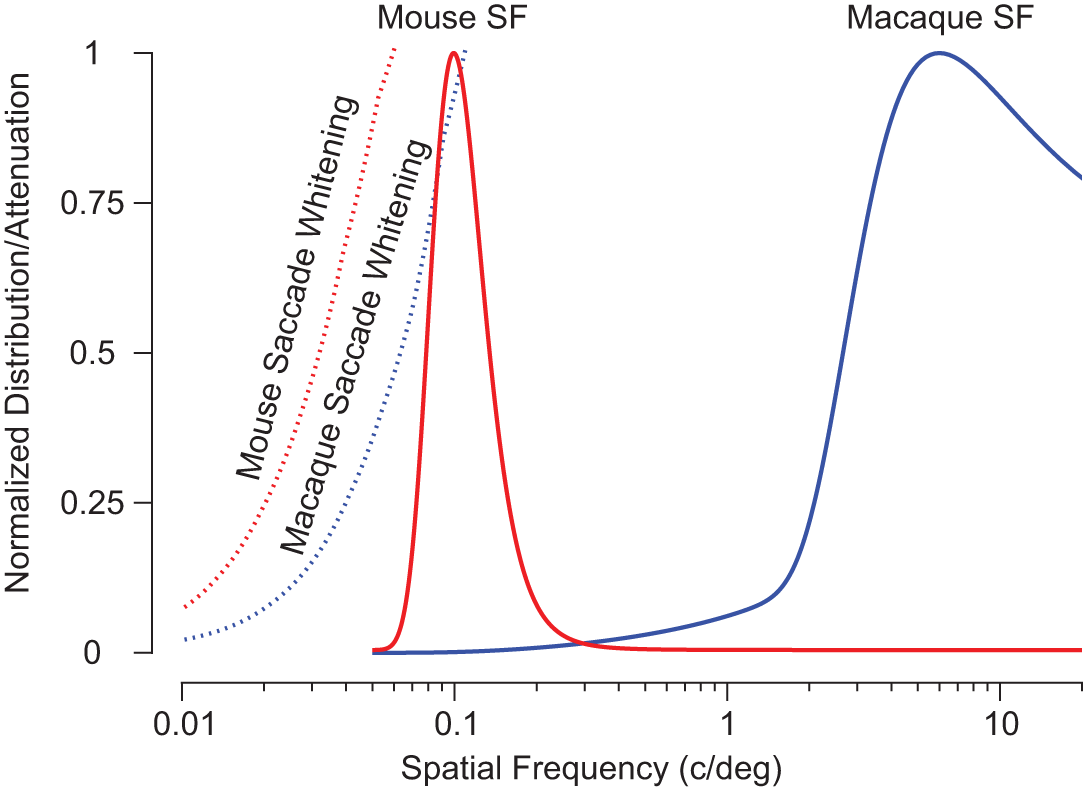

Supplement: Extended Data Figure 2-2 — Saccade and fixation dynamics whiten natural inputs to V1 Plots of spatial frequency distributions of model V1 receptive fields for mice (solid red) and macaques (solid blue) used to predict saccade sizes based on decorrelation of responses2 compared to the attenuation of spatial frequencies relative to natural images caused by >7-degree saccades (dashed red) and 3-4-degree saccades (dashed blue). We estimated attenuation by dividing the spectral data from Fig. 4C by the spectral density of natural images in Mostofi et al. (2020). Larger saccades and lower temporal frequencies shift the attenuation curve to lower spatial frequencies, while smaller saccades and higher temporal frequencies shift the attenuation to higher spatial frequencies. Download Figure 2-2, TIF file. [file eneuro-11-ENEURO.0287-23.2023-s008.tif]
